# Total Evidence, Average Consensus and Matrix Representation with Parsimony: What a Difference Distances Make

**Published:** 2007-02-13

**Authors:** Claudine Levasseur, François-Joseph Lapointe

**Affiliations:** 1 Département de sciences biologiques, Université de Montréal, QC, Canada

**Keywords:** Average consensus, character congruence, taxonomic congruence, matrix representation with parsimony, total evidence, simulation study

## Abstract

Matrix representation with parsimony (MRP) can be used to combine trees in the supertree or the consensus settings. However, despite its popularity, it is still unclear whether MRP is really a consensus method or whether it behaves more like the total evidence approach. Previous simulations have shown that it approximates total evidence trees, whereas other studies have depicted similarities with average consensus trees. In this paper, we assess the hypothesis that MRP is equally related to both approaches. We conducted a simulation study to evaluate the accuracy of total evidence with that or various consensus methods, including MRP. Our results show that the total evidence trees are not significantly more accurate than average consensus trees that accounts for branch lengths, but that both perform better than MRP trees in the consensus setting. The accuracy rate of all methods was similarly affected by the number of taxa, the number of partitions, and the heterogeneity of the data.

## Introduction

Matrix representation with parsimony (MRP) is certainly the most popular method to construct supertrees (Baum 1992; Ragan 1992), but it also applies in the consensus setting (sensu Bininda-Emonds 2003) when the input trees combined have the same leaf sets. Although MRP combines trees rather than the primary characters, Bininda-Emonds and Bryant (1998) discussed fundamental differences between MRP and consensus (namely, the ability of MRP to generate novel clades that were not implied by the input trees). Furthermore, the simulation study of Bininda-Emonds and Sanderson (2001) showed that MRP could be considered a good approximation of character combination (or total evidence, sensu Kluge 1989). Although Baum (1992) originally introduced MRP as a consensus method (see also Bryant 2003), and Pisani and Wilkinson (2002) showed that in the consensus setting MRP behave exactly as a consensus method is expected to behave, it is still unclear whether MRP trees are more similar to consensus or total evidence trees.

Interestingly, the differences between these alternative approaches are not as important as they seem when the data and trees are treated in a coherent fashion and when branch lengths are taken into account when combining trees (Lapointe et al. 1999). Indeed, Levasseur and Lapointe (2001) have shown that combining character or trees can provide very similar solutions, when using the average consensus (Lapointe and Cucumel 1997). More recently, Lapointe et al. (2003) further demonstrated that, in the consensus setting, there exists a close relationship between MRP and average consensus trees, when branch lengths are set to one. However, MRP has never been directly compared to the average consensus as a means of combining trees with branch lengths. In this paper, we investigate the similarities between total evidence (TE), MRP, and two variants of the average consensus (AC) that account for branch lengths or not when combining trees. With simulations, we compare the relative accuracy of the competing approaches in the consensus setting to assess whether MRP behaves more like consensus or total evidence.

## Methods

Model trees (MT) were generated with a Yule branching process using the program r8s (Sanderson 2003) and molecular sequences (or data partitions) of fixed lengths (2000 base pairs) were evolved on those trees using the program Seq-Gen (Rambault and Grassly 1997). The evolution of sequences was performed according to a Jukes-Cantor model (Jukes and Cantor 1969) with a site-to-site heterogeneity rate (shape parameter set to 0.5). To limit the number of simulations, we considered extreme cases only by fixing the number of taxa (*n*) to 10 or 30, and the number of data partitions (*k*) to 2 or 10. Homogeneous and heterogeneous data partitions were also generated for comparison purposes. To do so, the molecular sequences were respectively evolved on *k* trees with identical topologies and identical branch lengths (homogeneous data partitions), or *k* trees with identical topologies and branch lengths generated at random for a uniform [0–1] distribution (heterogeneous data partitions). For every combination of parameters (*n* and *k*), 1000 replicates were generated, and the molecular sequences evolved on the corresponding trees were treated as separate data partitions.

The data partitions were then analyzed either jointly or individually to estimate total evidence (TE) and separate trees alike. In all cases, a distance matrix was computed with a Jukes-Cantor model matching that used to generate the data, and an unweighted least-squares algorithm (Cavalli-Sforza and Edwards 1967) was employed to estimate trees with PAUP* (Swofford 1999). For the MRP analyses, the trees from separate data partitions were coded using RadCon (Thorley and Page 2000), and the resulting matrices were combined and analyzed with parsimony using PAUP* (see the protocol described in Bininda-Emonds and Sanderson 2001). When multiple equally parsimonious trees were obtained, the strict consensus of those trees was taken as the MRP solution. To compute average consensus (AC) trees, path-length distances were first extracted from the separate trees to compute an average distance matrix (program available upon request from the authors), and this average matrix was then analyzed using the same unweighted least-squares criterion (Cavalli-Sforza and Edwards 1967) that was used for the estimation of TE trees. In order to assess the effect of branch lengths, topological average consensus (TAC) trees were also computed by setting all branch lengths to one prior to the computation of the average distance matrix. Both variants of the average consensus were computed using the FITCH algorithm (with p = 0 and global rearrangements enabled) in PHYLIP (Felsenstein 1993).

The TE, MRP, AC, and TAC trees were compared with the model tree (MT) onto which the sequences were evolved to assess the performance of the competing approaches. Accuracy rates were obtained by counting the number of times that any given method recovered the correct MT topology, and ANOVA tests were computed to determine whether the rates of competing approaches were statistically different. Then, the different trees were compared with one another to assess the similarities among the various approaches. Topological identity (*Ti*) was measured by counting the number of times that any two methods produced identical trees, whereas topological similarity (*Ts*) was measured by computing the consensus fork index (Colless 1980) of the trees compared. A maximum value of one (1.0) is thus obtained for *Ts* when the two trees are identical and their strict consensus is fully resolved, whereas a null value (0.0) is obtained when their consensus is unresolved. Average values of *Ti* and *Ts* computed over the 1000 replicates are reported for comparison across methods.

## Results

Results of phylogenetic accuracy are reported in Table 1, for different numbers of taxa (*n*) and data partitions (*k*), as well as for homogeneous and heterogeneous data sets. These values indicate that TE always provides the best accuracy rates, whereas MRP always performs significantly worse than all other methods. Furthermore, AC that accounts for branch lengths outperforms TAC, providing results as good as TE except in the most extreme cases. Interestingly, both methods that ignore branch lengths (MRP and TAC) are also those that provide less resolved trees, thus explaining their poor accuracy rates. However, all approaches are affected identically by the number of taxa and data partitions. Increasing the number of taxa from 10 to 30 decreases accuracy rates, while increasing the number of data partitions from 2 to 10 increases accuracy rates. The best results are thus obtained for 10 taxa and 10 data partitions. In addition, the results for homogeneous data sets are always better than those based on heterogeneous data sets.

Table 2 presents the results of pairwise comparisons among TE, MRP, AC, and TAC trees. On average, the *Ti* index reveals that TE trees are more often identical to AC trees than they are to MRP trees. Furthermore, accounting for branch lengths does make a difference, as TAC and AC trees do not always produce trees with identical topologies. MRP trees seem to behave somewhat like TAC trees, especially for a larger number of data partitions. The *Ts* index further exhibits this trend for all methods and all conditions (Table 2). This similarity index reveals that even the most different methods (i.e. TE and MRP) have at least 80% of the nodes in common, in extreme cases. In general, the conclusions of these pairwise comparisons also mirror those obtained when comparing the competing trees to the model tree (Table 1). That is, that better results are obtained for more data partitions (*k* = 10), fewer taxa (*n* = 10), and homogeneous data.

## Discussion

In the present paper, we have assessed the accuracy and similarity of alternative approaches for treating separate data partitions in phylogenetic analysis. Using simulations, we evaluated the effect of the number of taxa, the number of partitions, and data heterogeneity to compare the performance of the competing approaches. We wanted to know whether MRP would behave more like a consensus method or like total evidence. Finally, we were interested in comparing consensus trees obtained by using actual branch length (AC), or by setting all branch lengths to one (TAC) prior to the computation.

Our results show that under the conditions investigated with simulations, the combined analysis of all data (TE) usually provide more accurate trees than separate analysis, regardless of the consensus method selected to combine trees. However, in most cases AC trees do almost as good as TE, and the results are only significantly better for a combined analysis in the most extreme cases (i.e. 30 taxa and 10 data partitions). On the other hand, MRP is always showing significantly worse accuracy rates than any other method. Moreover, accounting for branch lengths significantly improves the performance of AC trees with respect to TAC trees, except for one set of conditions (i.e. 10 taxa and 10 data partitions). These interesting results corroborate the study by Levasseur and Lapointe (2001) who already showed with actual data sets that total evidence and consensus can produce identical results when treated in a coherent fashion, using the average consensus (see also Lapointe et al. 1999).

Our results also show that the different approaches are affected in the same way by the parameters of the simulations. Better accuracy rates are always obtained with more partitions, fewer taxa, and homogeneous data, in agreement with the conclusions of other simulation studies (Bininda-Emonds and Sanderson 2001; Bininda-Emonds 2003), and other theoretical works (Erdos et al. 1999, Moret et al. 2002). For one, adding more characters increases the number of informative sites, which also increases the phylogenetic signal. For that matter, all simulations based on 10 partitions (20000 characters) provided much better results than those based on 2 partitions (4000 characters). On the other hand, using fewer taxa reduces the number of possible trees, and this also decreases the probability of estimating the wrong tree. Consequently, the worst results are obtained for 30 taxa and 2 data partitions. In such situations, the best method recovers the correct MT topology in only 10% of the replicates generated. Finally, data heterogeneity also affects accuracy by decreasing the phylogenetic signal. Although our simulations were based on model trees with identical topologies, randomizing the branch lengths had a strong negative impact on the accuracy rates. Previous simulations (Levasseur and Lapointe 2003) have also shown that changing the evolutionary rate of data partitions decreases accuracy. In practice, this problem is likely to be even worse when data partitions with incompatible phylogenetic histories are combined.

The negative effect of the number of taxa on accuracy is not comforting, since the methods that are compared in this paper were also developed to construct large supertrees. Bininda-Emonds and Sanderson (2001) observed that a reduction of the overlap among the trees combined greatly decreases accuracy. When heterogeneous data partitions representing overlapping sets of leaves are combined (i.e. in the supertree setting), this effect is even more dramatic (Lapointe and Levasseur 2004), and such conditions are likely to jeopardize the quest for the Tree of Life. Still, one strong argument for supertree methods (including MRP) is that it ought to be faster to build several small trees and piece them together than to compute one large tree (see Sanderson and Driskell 2003), given the computational complexity of the optimization problems.

We have already shown (Levasseur and Lapointe 2001) that average consensus trees are more similar to total evidence trees than those derived from consensus methods that ignore branch lengths (e.g. strict, majority rule, Adams). The present study corroborates these results by showing that accounting for branch lengths makes a difference, even when the same consensus method is employed. We also show that MRP trees are further from total evidence trees than either form of the average consensus. Furthermore, when actual branch lengths are ignored in the computation, average consensus trees become increasingly similar to MRP trees, as predicted by Lapointe et al. (2003).

Last but not least, contrary to the claim of Bininda-Emonds and Bryant (1998), our simulations clearly showed that MRP does not behave like total evidence. There seems to be similarities between MRP and average consensus trees when all branch lengths are set to one, although the consensus approach always outperforms MRP in terms of accuracy. In their paper comparing MRP with several topological consensus methods, Bininda-Emonds and Sanderson (2001) suggested that MRP trees can produce novel clades that are contradicted by some or all of the input trees. Average consensus trees also share this property (Wilkinson et al. 2005). The joint use of average consensus and total evidence has been proposed to increase phylogenetic accuracy in such cases (Lapointe et al. 1999). Further studies should now evaluate the relationships between super-tree methods that account for branch lengths (e.g. Criscuolo et al. 2006) and weighted version of MRP that accounts for bootstrap support values (Ronquist 1996), with respect to the analysis of supermatrices in a phylogenomic framework (Delsuc et al. 2005).

## Figures and Tables

**Table 1:** Accuracy rates of total evidence (TE), average consensus (AC), topological average consensus (TAC), and matrix representation with parsimony (MRP) under different simulation parameters. The different letters are associated to the methods that are significantly different from the others in each set of simulations. *n* = number of taxa; *k* = number of data partitions.

	Homogeneous data		Heterogeneous data	

*n* = 10	*k* = 2	*k* = 10	*k* = 2	*k* = 10
	
TE	589 a	787 a	538 a	768 a
AC	572 a	761 ab	511 a	751 ab
TAC	477 b	730 b	419 b	729 b
MRP	303 c	668 c	247 c	655 c
*n* = 30	*k* = 2	*k* = 10	*k* = 2	*k* = 10
TE	85 a	333 a	67 a	317 a
AC	72 a	265 b	54 a	261 b
TAC	26 b	187 c	20 b	223 c
MRP	6 c	118 d	2 c	125 d

**Table 2:** Results of pairwise comparisons of total evidence (TE), average consensus (AC), topological average consensus (TAC), and matrix representation with parsimony (MRP). Topological identity (*Ti*) values are reported, as well as topological similarity (*Ts*) values (in parentheses). *n* = number of taxa; *k* = number of data partitions.

	Homogeneous data		Heterogeneous data	

*n* = 10	*k* = 2	*k* = 10	*k* = 2	*k* = 10
	
TE - AC	778 (0.964)	870 (0.979)	731 (0.954)	857 (0.977)
TE - TAC	606 (0.931)	804 (0.969)	525 (0.914)	802 (0.968)
TE - MRP	359 (0.859)	721 (0.953)	293 (0.837)	702 (0.947)
AC - TAC	637 (0.938)	851 (0.978)	566 (0.925)	845 (0.977)
AC - MRP	359 (0.860)	745 (0.957)	295 (0.839)	735 (0.954)
TAC - MRP	359 (0.860)	816 (0.969)	297 (0.839)	785 (0.962)
*n* = 30	*k* = 2	*k* = 10	*k* = 2	*k* = 10
TE - AC	309 (0.953)	505 (0.974)	279 (0.946)	427 (0.968)
TE - TAC	86 (0.914)	321 (0.959)	63 (0.902)	327 (0.957)
TE - MRP	10 (0.831)	183 (0.935)	5 (0.812)	173 (0.932)
AC - TAC	134 (0.928)	456 (0.971)	97 (0.921)	467 (0.971)
AC - MRP	11 (0.832)	221 (0.941)	5 (0.815)	211 (0.940)
TAC - MRP	10 (0.834)	294 (0.949)	5 (0.816)	265 (0.947)
